# CCN2 mediates fibroblast-macrophage interaction in knee arthrofibrosis based on single-cell RNA-seq analysis

**DOI:** 10.1038/s41413-025-00400-9

**Published:** 2025-02-24

**Authors:** Ziyun Li, Jia Jiang, Kangwen Cai, Yi Qiao, Xuancheng Zhang, Liren Wang, Yuhao Kang, Xiulin Wu, Benpeng Zhao, Xiuli Wang, Tianyi Zhang, Zhiqi Lin, Jinlong Wu, Simin Lu, Haihan Gao, Haocheng Jin, Caiqi Xu, Xiaoqiao Huangfu, Zhengzhi James, Qiuhua Chen, Xiaoqi Zheng, Ning-Ning Liu, Jinzhong Zhao

**Affiliations:** 1 Department of Sports Medicine, Department of Orthopedic Surgery, Shanghai Sixth People’s Hospital Affiliated to Shanghai Jiao Tong University School of Medicine, Shanghai, 200233 China; 2 Shanghai Normal University, Shanghai, 200233 China; 3 State Key Laboratory of Systems Medicine for Cancer, Center for Single-Cell Omics, School of Public Health, Shanghai Jiao Tong University School of Medicine, Shanghai, 200025 China; 4 Center for Single-Cell Omics, School of Public Health, Shanghai Jiao Tong University School of Medicine, Shanghai, 200025 China

**Keywords:** Diseases, Physiology

## Abstract

Knee arthrofibrosis, characterized by excessive matrix protein production and deposition, substantially impairs basic daily functions, causing considerable distress and financial burden. However, the underlying pathomechanisms remain unclear. Here, we characterized the heterogeneous cell populations and cellular pathways by combination of flow cytometry and single-cell RNA-seq analysis of synovial tissues from six patients with or without knee arthrofibrosis. Increased macrophages and fibroblasts were observed with decreased numbers of fibroblast-like synoviocytes, endothelial cells, vascular smooth muscle cells, and T cells in the arthrofibrosis group compared with negative controls. Notably, fibroblasts were discovered to interact with macrophages, and lead to fibrosis through TGF-β pathway induced *CCN2* expression in fibroblasts. CCN2 was demonstrated to be required for fibroblast pro-fibrotic functions (activation, proliferation, and migration) through TGFBR/SMAD pathway. The expression of CCN2 was positively correlated with the collagen volume and TGF-β expression and negatively associated with patient-reported outcome measures in another cohort of patients with knee arthrofibrosis. Our study reveals the role of CCN2 in the fibroblast-macrophage interaction through TGF-β pathway which might help to shed light on CCN2 as a potential biomarker.

## Introduction

Knee arthrofibrosis (AF) is characterized by a limited range of motion (ROM) in active knee extension and/or flexion, typically not caused by infection or other specific factors.^1^ It is a common post-operative complication, with an incidence of 2%–35% following anterior cruciate ligament (ACL) reconstruction and 0.2%–10% after total knee replacement.^2–4^ Nearly 3 million patients undergo AF procedures annually in the USA.^5^ Despite the considerable progress in arthrolysis surgeries (arthroscopic surgeries^6,7^ and open surgeries^8,9^), the treatment for knee AF remains unsatisfactory. Patients with knee AF experience progressive loss of daily function, running and squatting ability, and eventually become incapable of sitting or climbing the stairs, resulting in considerable agony and a huge financial burden (increased hospitalization cost of more than $7 000 compared with a primary surgery for total knee replacement).^10–12^ Therefore, researches are in great need to prevent and treat knee AF.^1^

Pathologically, knee AF usually arises from surgeries or injuries, leading to the intricate disruption of innate and adaptive immune responses, which play crucial roles in the pathogenesis of numerous chronic inflammatory disorders.^13^ The activation of immune cells (including macrophages, T cells, and mast cells) by these factors signals the initiation of the following pro-fibrotic cascade.^13^ Specifically, pro-fibrotic cytokines can affect fibroblasts by disrupting the balance between extracellular matrix (ECM) production and degradation, resulting in the excessive deposition of collagenous and non-collagenous matrix proteins, especially type I collagen (COL1).^14^ With the alteration in composition accompanied by extensive cross-linking of the ECM, it becomes highly resistant to degradation, resulting in knee AF.^13,15^ Currently, multiple biological pathways and biomarkers have been identified by immunostaining and bulk RNA-seq within fibroblasts including the PI3K/AKT/mTOR pathway,^16^ TGFβ/SMAD pathway,^13^ MMPs,^17^ ADAMTS,^17^ TIMPs,^17^ xylosyltransferases I and II,^18,19^ TGFBR1,^20^ CD68,^21^ β-catenin,^21^ BMP-2,^22^ and ITGA1.^15^ However, all these studies were only at the preclinical stage, mainly because they were unable to illustrate the complex cellular landscape of knee AF and elucidate the specific molecular mechanisms due to technical limitations. Consequently, the exact pathogenic mechanism of knee AF remains unknown, resulting in a lack of reliable biomarkers and therapeutic targets for knee AF.

In this study, we reveal the heterogenic cell populations of synovial tissues in knee AF by a combination of flow cytometry (FCM) and single-cell RNA sequencing (scRNA-seq) analysis. Our findings highlight the co-existence of fibroblast-macrophage interactions through TGF-β pathway and the autocrine fibroblast circuit of CCN2 in knee AF by both in vitro and in silico experiments. We then identified CCN2 as a potential biomarker for knee AF through immunostaining in another patient cohort.

## Results

### A comprehensive single-cell atlas of human synovial tissue by FCM and scRNA-seq analysis

**Fig. 1:**
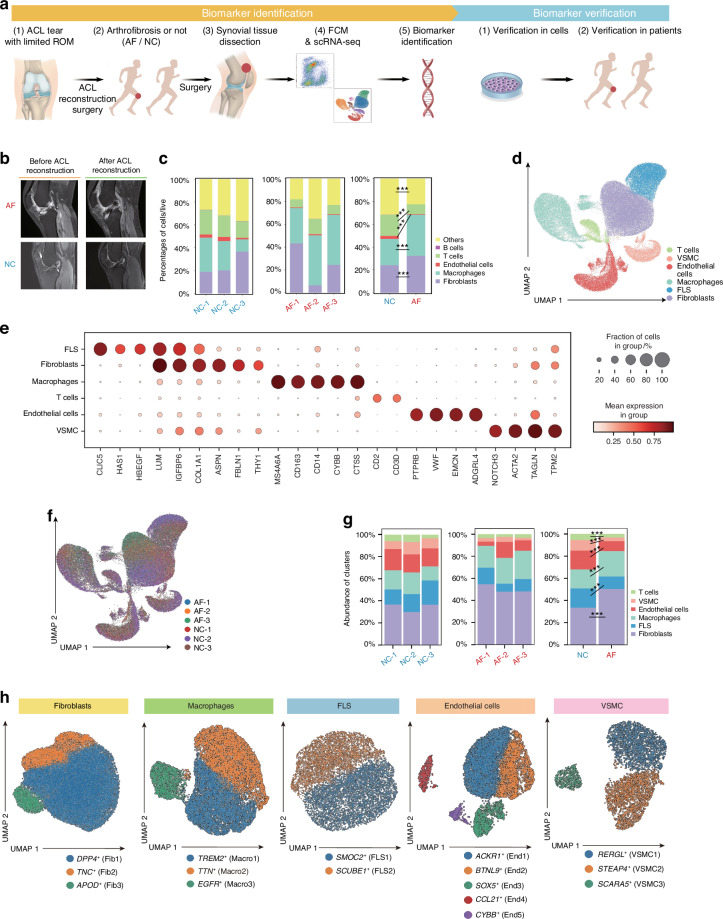
A comprehensive single-cell atlas of human synovial tissue by FCM and scRNA-seq analysis. **a** Schematic workflow of the experimental strategy, including “Biomarker Identification” and “Biomarker Verification.” Patients with ACL injury and loss of range of motion were treated properly in the ACL reconstruction surgery. After the initial surgery, patients underwent a year of rehabilitation were recruited, and those with knee arthrofibrosis were allocated in the AF group (arthrofibrosis) while others were allocated in the NC group (negative controls). Synovial tissues were obtained during the second surgery and digested to single-cell suspension. FCM and scRNA-seq analysis were performed to identify the biomarker of knee arthrofibrosis, which was further verified in cell lines and clinical patients. **b** Representative pre-reconstruction and post-reconstruction MRI figures of patients in AF and NC groups. **c** Percentages of major synovial cell types assesed by flow cytometry. **d** Cell clusters across 68 144 cells (six donors) shown on a UMAP visualization colored according to cell types. **e** Bubble plot of clustering analysis revealing six cell clusters defined in (**d**) with scaled expression of differentially expressed genes. **f** Cell clusters from six donors shown on a UMAP visualization colored by donors. **g** Cluster abundance of synovial tissue by scRNA-seq analysis. **h** UMAP plots showing subpopulations of fibroblasts, macrophages, FLS, endothelial cells, and VSMC by scRNA-seq. ACL, anterior cruciate ligament; FCM, flow cytometry; scRNA-seq, single-cell RNA sequencing; UMAP, uniform manifold approximation and projection; FLS, fibroblast-like synoviocytes; VSMC, vascular smooth muscle cells; ***P < 0.001

To elucidate the mechanism underlying knee AF, we performed FCM and scRNA-seq analysis on human synovial tissues surgically resected from patients with AF (AF group) or negative controls (NC group) (Fig. 1a). The baseline information of all six patients in both groups (AF, *n* = 3; NC, *n* = 3) is listed in Table S1. Representative magnetic resonance imaging (MRI) images of patients in both groups are presented in Fig. 1b. The MRI images reveals widespread heterogeneous thickening of the synovium (fibrotic synovial tissue) in AF after ACL reconstruction, but no thickening in NC.^23^

By FCM analysis, we observed that T cells, endothelial cells, macrophages, and fibroblasts were the main components of the synovial tissue (Fig. S1). Specifically, PDPN were used to mark synovial fibroblasts(Fig. S1). Interestingly, more macrophages and fibroblasts, and fewer T cells and endothelial cells, were observed in AF compared with NC (*P* < 0.001) (Fig. 1c).

Next, we performed scRNA-seq analysis of the three AF and three NC samples. After quality control, we obtained a total of 68,144 cells that can be clustered into six distinct cell groups, respectively annotated as fibroblasts (characterized by *THY1* and *LUM*^24,25^), macrophages (characterized by *CD14* and *MS4A4A*^26,27^), fibroblast-like synoviocytes (FLS), endothelial cells, vascular smooth muscle cells (VSMC), and T cells (Fig. 1d, e). All cell types were shared across the six patients (Fig. 1f). Consistent with the FCM results, we observed an increase in macrophages and fibroblasts in AF samples compared with NC samples (*P* < 0.001) (Fig. 1g). The cell subpopulations of fibroblasts, macrophages, FLS, endothelial cells, and VSMC are further illustrated in Fig. 1h. Subsequent analyses mainly focused on the macrophages and fibroblasts.

Overall, both FCM and single-cell transcriptional analyses indicated a strong relationship between AF and enrichment of both macrophages and fibroblasts.

### Distinct fibrotic signatures of human synovial fibroblasts

Fibroblasts, located in the sublining layer of the knee synovium, are key effector cells in knee AF.^24,28,29^ Normally, they produce ECM to form the structural skeleton of tissues and maintain adhesion and ECM release-reabsorption balance.^14^ However, in fibrotic diseases, fibroblasts produce excessive ECM and promote fibrosis by inducing cytokine secretion.^30^

**Fig. 2:**
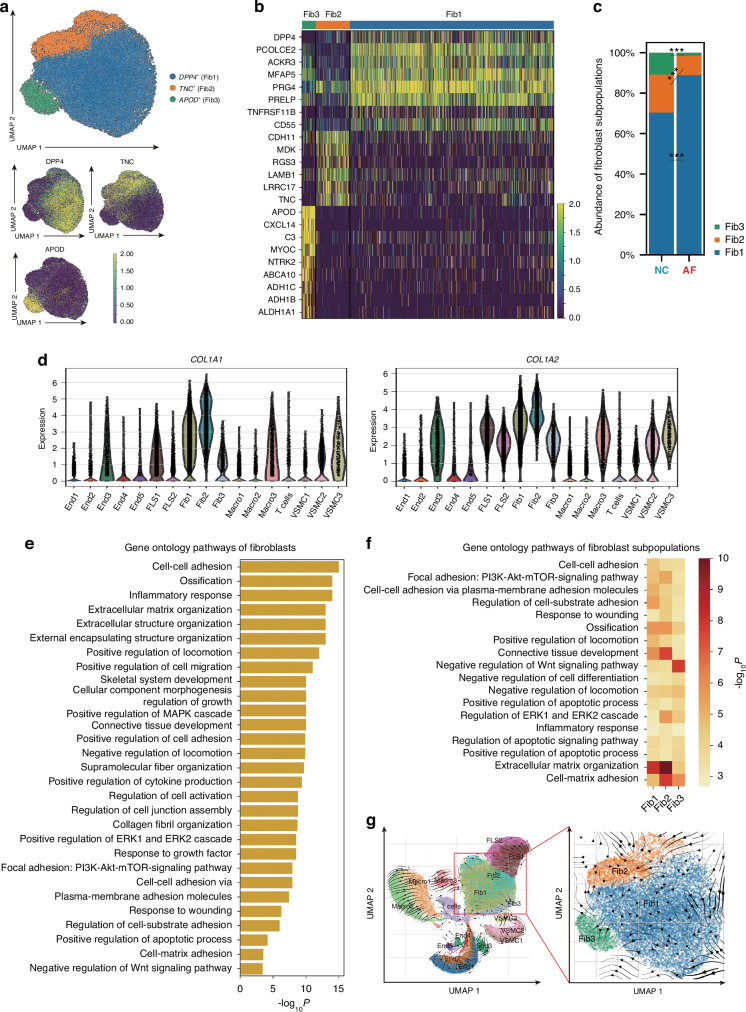
Distinct fibrotic signatures of human synovial fibroblasts. **a** UMAP analysis of three subpopulations of fibroblasts: *DPP4*^*+*^ fibroblasts (Fib1), *TNC*^*+*^ fibroblasts (Fib2), and *APOD*^*+*^ fibroblasts (Fib3) by scRNA-seq. **b** Transcriptional profile of three subpopulations of fibroblasts are shown in the heatmap. **c** Abundance of fibroblast subpopulations in the AF and NC groups by scRNA-seq. **d** Violin plot showing the expression of *COL1A1* and *COL1A2* mRNA among all subpopulations. **e** Bar plot showing GO analysis for fibroblasts. **f** Heatmap showing GO analysis across fibroblasts subpopulations. **g** RNA velocity analysis indicating cell differentiation of fibroblasts from three subpopulations. UMAP, uniform manifold approximation and projection; ***P < 0.001

Using single-cell transcriptome analysis, we identified three putative subpopulations of 28,134 fibroblasts: *Dipeptidyl peptidase-4* (*DPP4*)^*+*^ fibroblasts (Fib1), *Tenascin C* (*TNC*)^*+*^ fibroblasts (Fib2), and *Apolipoprotein D* (*APOD*)^*+*^ fibroblasts (Fib3) (Fig. 2a, b). By comparing the proportion of fibroblast subpopulations between the AF and NC groups, we discovered that more Fib1 were observed in the AF group than that in the NC group, whereas the proportion of Fib3 decreased in the AF group (*P* < 0.01) (Fig. 2c).

Next, we explored the cells that were activated myofibroblasts, the main contributors to fibrosis expressing the most ECM genes.^31^ As illustrated in Fig. 2d, the transcriptional expression of *COL1A1* and *COL1A2* in Fib1 and Fib2 was the highest among all subpopulations in the synovial tissues. COL1, a type of collagen with high tensile strength, is recognized as the main component of the ECM in fibrotic diseases.^14^ A higher COL1/elastin ratio was also identified in fibrotic tissues than in healthy tissues.^14^ Thus, we suggested that Fib1 and Fib2 were myofibroblasts while Fib3 were quiescent fibroblasts. We then performed Gene Ontology (GO) analysis, a computational method used in bioinformatics to understand the biological functions of genes and their products. We found that pathways related to fibrosis, such as cell-cell adhesion and extracellular matrix organization analysis were highly enriched in fibroblasts (Fig. 2e). Especially, such pro-fibrotic pathways were prominently enriched in Fib1 and Fib2, whereas Fib3 exhibited relatively high enrichment in anti-fibrosis pathways, such as negative regulation of Wnt (Fig. 2f). Together, these results revealed that Fib1 and Fib2 could be the major effector cells in fibrosis, whereas Fib3 may be hardly involved in this process. Our findings are consistent with those of previous studies on the functions of these fibroblasts.^32–36^ The transcriptome of the Fib1 cluster is characterized by *DPP4*, which is well-established as a pro-fibrotic gene.^36^ For Fib2, fibroblasts highly expressing *TNC* and *CDH11* are believed to strongly promote fibrosis and greatly contribute to the migration and proliferation of fibroblasts.^32–34^ Additionally, Fib3 characterized by the elevated expression of *APOD*, *C3*, and *CXCL14* in knee synovial tissues have been identified previously.^35^ This subset of fibroblasts has been demonstrated to be less relevant to fibrosis.^35^

To investigate the differentiation streams among these three subpopulations, we performed RNA velocity analysis, an approach that retrieves information regarding the direction of a single cell within the transcriptional space by detecting unspliced and spliced mRNAs.^37^ The three subpopulations of fibroblasts were ordered in a pseudotemporal manner (on a scale of hours). The direction of cell differentiation and the extent of transcriptional changes are indicated by the vector (arrow) direction and length, respectively.^37^ As depicted in Fig. 2g, we observed two differentiation streams of fibroblasts: progression from Fib1 to Fib2 and from Fib1 to Fib3. This suggests that the differentiation streams are mainly fueled by Fib1, consistent with a previous report revealing that *DPP4*^*+*^ fibroblasts are progenitor cells.^38^ Collectively, we identified three subpopulations of fibroblasts in synovial tissue and discovered that *DPP4*^*+*^ and *TNC*^*+*^ fibroblasts were closely related to knee AF.

### Identification and molecular characterization of synovial macrophages and other synovial cells

**Fig. 3:**
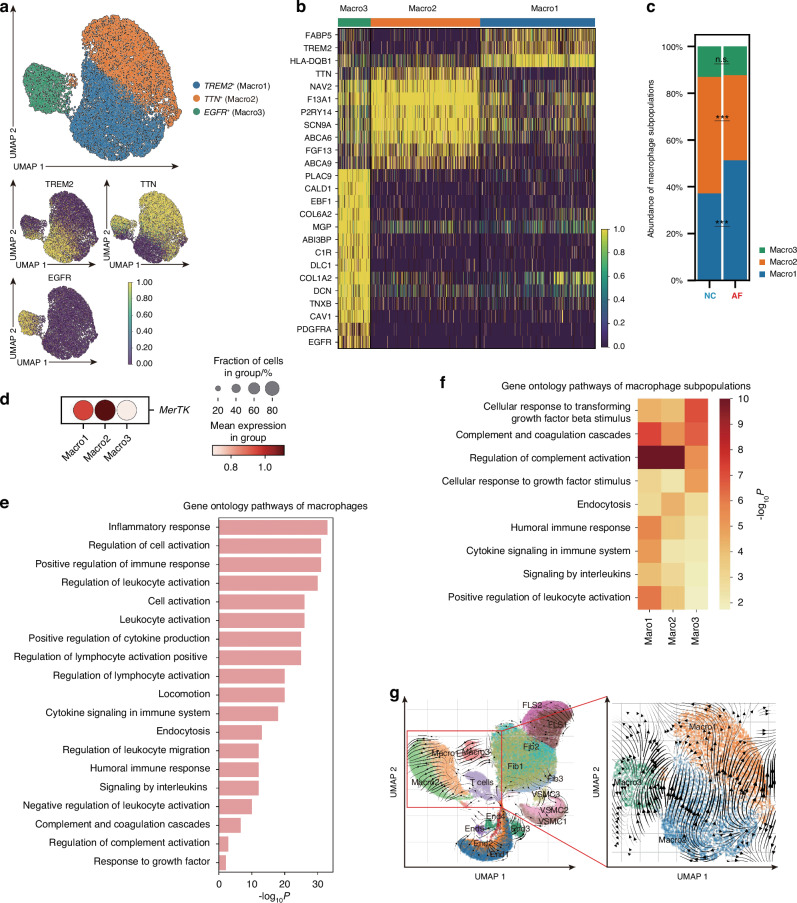
Identification and molecular characterization of synovial macrophages. **a** UMAP of three subpopulations of macrophages: *TREM2*^*+*^ macrophages (Macro1), *TTN* ^*+*^ macrophages (Macro2) and *EGFR*^*+*^ macrophages (Macro3) by scRNA-seq. **b** Transcriptional profile of three subpopulations of macrophages shown on a heatmap. **c** Abundance of macrophage subpopulations between the AF group and the NC group by scRNA-seq. **d** Bubble plot showing expression of *MerTK* in Macro1, Macro2, and Macro3. **e** Bar plot showing GO analysis for macrophages. **f** Heatmap of GO analysis for each subpopulation indicating different functions. **g** RNA velocity analysis indicating cell differentiation of macrophages from three subpopulations. UMAP, uniform manifold approximation and projection; ***P < 0.001; n.s., no significance

As an important source of pro-fibrotic factors, including TGF-β, IL-1β, and PDGF, macrophages are important in knee AF^13,39^ and are highly plastic and heterogeneous.^40^ Our single-cell transcriptome analysis identified three macrophage subpopulations: *Triggering receptor expressed on myeloid cells 2* (*TREM2*)^*+*^ macrophages (Macro1), *Titin* (*TTN*)^+^ macrophages (Macro2), and *Epidermal growth factor receptor* (*EGFR*)^*+*^ macrophages (Macro3) (Fig. 3a, b). We then compared the composition of macrophage subpopulations between the AF and NC groups and observed increased Macro1 (*P* < 0.001) and decreased Macro2 in the AF group (*P* < 0.001), whereas the proportion of Macro3 remained unaltered in both groups (*P* = 0.247) (Fig. 3c).

To examine the function of macrophages, two groups of macrophages were identified: *MerTK*^*+*^ macrophages lying in the lining layer of the synovial tissue and *MerTK-* ones lying in the sublining layer.^40,41^ We demonstrated that *MerTK* mRNA was expressed in subpopulations of macrophages. Compared with Macro3, Macro1 and Macro2 highly expressed *MerTK* (Fig. 3d). Thus, we identified Macro1 and Macro2 as the lining macrophages while Macro3 as the sublining macrophages. Regarding the traditional classification of macrophages (M1 macrophages and M2 macrophages), we found that Macro1 and Macro2 highly expressed M1 markers and Macro3 highly expressed M2 marker (Fig. S2). Thus, Macro1 and Macro2 can be considered as M1 macrophages while Macro3 as M2 macrophage. Based on GO analysis, we discovered high enrichment of immune responses and cell signalling in macrophages (Fig. 3e). Interestingly, GO analysis of these subpopulations revealed great functional diversity among the three subpopulations. Macro1 and Macro2 were highly enriched in immune processes, including the regulation of complement activation, humoral immune response, positive regulation of leukocyte activation, regulation of complement activation, and endocytosis (Fig. 3f). In contrast, enrichment of GO terms, including cellular response to transforming growth factor beta stimulus and cellular response to growth factor stimulus, were observed in Macro3 (Fig. 3f). This suggests that Macro1 and Macro2 are more involved in innate immune responses, whereas Macro3 is highly associated with cellular communication. Consistent with previous studies, the lining macrophages (*MerTK* ^+^ ) exhibited multiple homeostatic functions and are involved in antimicrobial activity.^41^ In contrast, the sublining macrophage Macro3 in our study highly expressed pro-fibrotic genes, such as *EGFR* and *PDGFRa*, greatly involved in cell communication, indicating that Macro3 might contribute to communication with fibroblasts, playing an important role in the fibrosis process.^41,42^

RNA velocity analysis was then performed to investigate the transcriptional status of the three macrophage subpopulations. Considering the results of RNA velocity and GO analysis, Macro1 was more likely to differentiate toward Macro2, while Macro3 was more static and thus identified as a distinct cluster of macrophages different from Macro1 and Macro2 (Fig. 3g). Thus, Macro3 may be involved in fibrosis. Collectively, we observed high heterogeneity within macrophages in synovial tissues, and Macro3 may contribute to fibrosis.

FLS, a synovial-specific cell type, also plays an important role in pathological conditions such as osteoarthritis and rheumatoid arthritis.^43–45^ We identified two major subpopulations of FLS: *SPARC related modular calcium binding 2* (*SMOC2*) ^+^ FLS (FLS1) and *Signal peptide, cub domain and EGF like domain containing 1*(*SCUBE1*) ^+^ FLS (FLS2) (Fig. S3A, B). A slight increase in FLS2 expression was observed in the AF group compared with that in the NC group (Fig. S3C). Gene enrichment analysis revealed subtle differences, indicating that FLS2 contributed more to the cellular response to fluid shear stress and elastic fiber formation, and both subpopulations were not highly involved in fibrosis-related terms (Fig. S3D). Further investigation of the putative transcriptional states of FLS1 and FLS2 is presented in Fig. S3E, suggesting differentiation from FLS2 to FLS1.

Regarding blood vessel-related cells, endothelial cells and VSMC were identified in the synovial tissues. Endothelial cells were clustered into five subpopulations based on research by Ramachandran et al.^42^: *Atypical chemokine receptor 1* (*ACKR1*)^*+*^ endothelial cells (End1), *Butyrophilin like 9* (*BTNL9*)^*+*^ endothelial cells (End2), *SRY-box transcription factor 5* (*SOX5*)^*+*^ endothelial cells (End3), *Chemokine (C-C motif) ligand 21* (*CCL21*)^*+*^ endothelial cells (End4) and *Cytochrome b-245 beta chain* (*CYBB*)^*+*^ endothelial cells (End5) (Fig. S4A, B). We then explored the proportions of different endothelial cell subpopulations in both groups (Fig. S4C). We further investigated the functions of all the subpopulations. By GO analysis, the response to mechanical stimulus and regulation of endothelial cell proliferation were enriched in End1, blood vessel development was more enriched in End2, End3 and End4 exhibited high enrichment in the extracellular matrix organization, and an apparent enrichment of inflammatory response was observed in End5 (Fig. S4D). The relationship among these subpopulations and the differentiation streams was analyzed using RNA velocity (Fig. S4E), suggesting that the differentiation streams may be highly related to the different functions of these subpopulations.

VSMC, located in the medial layer of arteries, is responsible for arterial contraction and ECM production, including elastins, collagens, and proteoglycans.^46^ Three subpopulations of VSMC were discovered: *Ras-related and estrogen-regulated growth inhibitor-like protein* (*RERGL*) ^+^ VSMC (VSMC1), *Six transmembrane epithelial antigen of prostate 4* (*STEAP4*) ^+^ VSMC (VSMC2) and *Scavenger receptor class A member 5* (*SCARA5*) ^+^ VSMC (VSMC3) (Fig. S5A, B). A decrease in VSMC2 levels was observed between the two groups (*P* < 0.001) (Fig. S5C). GO analysis revealed that the circulatory system process and cellular response to growth factor stimulus, the PI3K-Akt signaling pathway, and other biological processes were enriched in VSMC1, whereas extracellular structure organization and ECM organization processes were more enriched in VSMC2 and VSMC3 had a high enrichment in blood vessel development and extracellular structure organization processes (Fig. S5D). The differentiation streams of the three subpopulations are presented in Fig. S5E, indicating that VSMC2 is terminally differentiated.

### Identification of *CCN2* as a biomarker of knee AF based on fibroblast-macrophage interactions

**Fig. 4:**
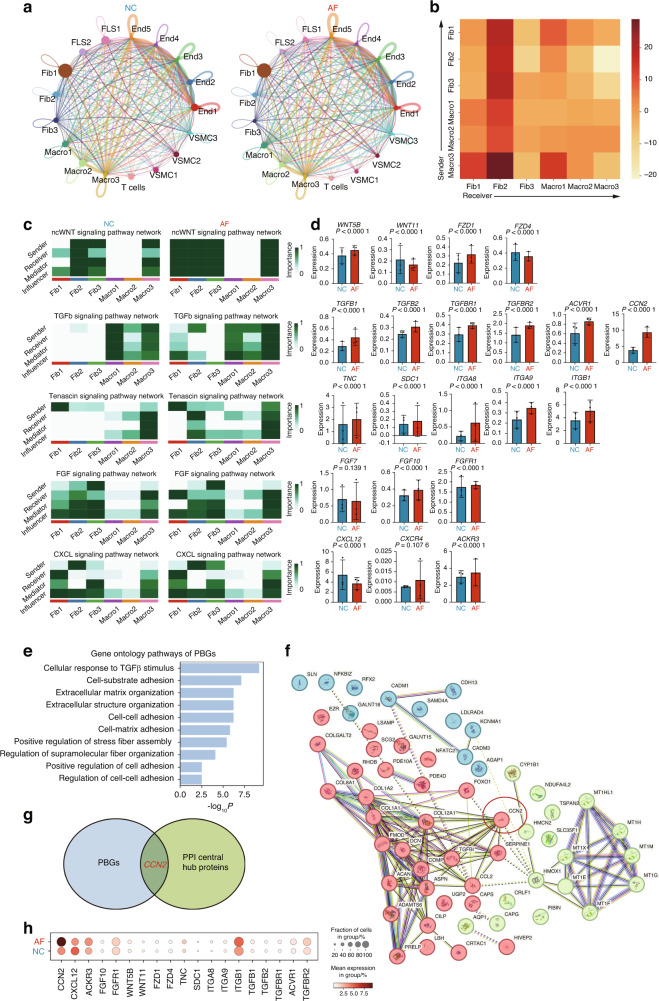
Identification of CCN2 as a biomarker of knee arthrofibrosis based on fibroblast-macrophage interactions. **a** The signaling networks in AF and NC. Circle sizes are proportional to the number of cells in each cell group, and edge width represents the interaction probability. **b** Heatmap of pathway changes between AF and NC. The redder the color block in the figure, the more interaction counts in the AF group. **c** Heatmap of the relative contribution of fibroblasts and macrophages to a specific signaling pathway network. **d** Pathway-related gene expression in fibroblasts within the AF group and the NC group. **e** Bar plot showing GO analysis for PBGs. **f** Protein-protein interaction (PPI) network of top 50 DEGs. Three clusters were identifitied (coloured red, blue and green), and CCN2 were highlighted in a red circle. **g** Venn diagram of PBGs overlapped with PPI central hub proteins. **h** Bubble plot of expression of PBGs in all cells involved in the AF and NC groups. PBG, potential biomarker gene. Data are shown as mean ± SD

We further explored the interaction between cells using CellChat to predict cell-cell interactions.^47^ We found a high interaction probability between subpopulations in endothelial cells, endothelial cells and VSMCs and immune cells and non-immune cells (macrophages and fibroblasts, macrophages and endothelial cells) in both AF and NC (Fig. 4a). Comparing with NC, we found a dramatic increase in fibroblast-macrophage interaction in AF. Based on our results that the percentages of fibroblasts and macrophages in synovial tissues significantly increased in the AF group, which was closely related to fibrosis via ECM production and stimulus responses, we further focused on the fibroblast-macrophage interaction. The fibroblast-macrophage interaction has been well established in other fibrotic diseases as driving factors and is considered as a conserve mechanism across organs, namely pulmonary fibrosis, renal fibrosis and liver fibrosis.^39,48,49^ We then compared the interactions of these two specific cell types in the AF group with those in the NC group (Fig. 4b). We observed that Fib1 and Fib2 mainly served as receiving cells, while Macro1, Macro2, and Macro3 mainly served as sending cells. For further investigation, we selected the pro-fibrotic pathways, including the non-canonical Wnt (ncWnt), TGF-β, tenascin, FGF, and CXCL signaling pathways. In general, these pathways were upregulated in Macro3, Fib1, and Fib2 cells in the AF group (Fig. 4c). Specifically, in the CXCL2 pathway, fibroblasts are more likely to be active as senders, while macrophages tend to be receivers, mainly because fibroblasts participate in the recruitment of macrophages and produce pro-fibrotic effects in this pathway.^50^ In addition, fibroblasts were more likely to act as receivers, whereas macrophages were senders during interactions through the other four different pathways, which are all pro-fibrotic (Fig. 4c).^34,39,51–53^ This result aligns with the observation that Fib1 and Fib2 were mainly involved in fibrotic processes, and Macro3 was more involved in intercellular communication.

To further investigate on the molecular mechanism and the potential biomarker, we focused on the changes in pathway-related genes in fibroblasts and identified 21 genes in pro-fibrotic pathways (Fig. 4d). Based on statistical differences, 19 genes were then regarded as potential biomarker genes (PBGs). As expected, GO analysis revealed high enrichment of pro-fibrotic pathways in PBGs (Fig. 4e). Additionally, we performed STRING protein-protein interaction (PPI) of the top 50 differentially expressed genes (DEGs) of fibroblasts between AF and NC (Table S2). This PPI network consisted of 3 major clusters and multiple central hub proteins (Fig. 4f). CCN2 was identified to be in both PBGs and central hub proteins (Fig. 4g). Then, we compared the expression of PBGs in all cells from both groups. *CCN2* expression in the AF group was significantly higher than that in the NC group (Fig. 4h), which was also among the top five DEGs of fibroblasts (Table S2). Thus, CCN2 was considered to be a potential biomarker for knee AF.

In general, we demonstrated active fibroblast-macrophage interactions in knee AF and multiple pro-fibrotic pathways. Particularly, *CCN2* in the TGF-β pathway expressed by fibroblasts was proposed as a potential biomarker of knee AF.

### Knockdown of *CCN2* attenuated the pro-fibrotic functions of fibroblasts by downregulating TGFBR/SMAD signalling pathway

**Fig. 5:**
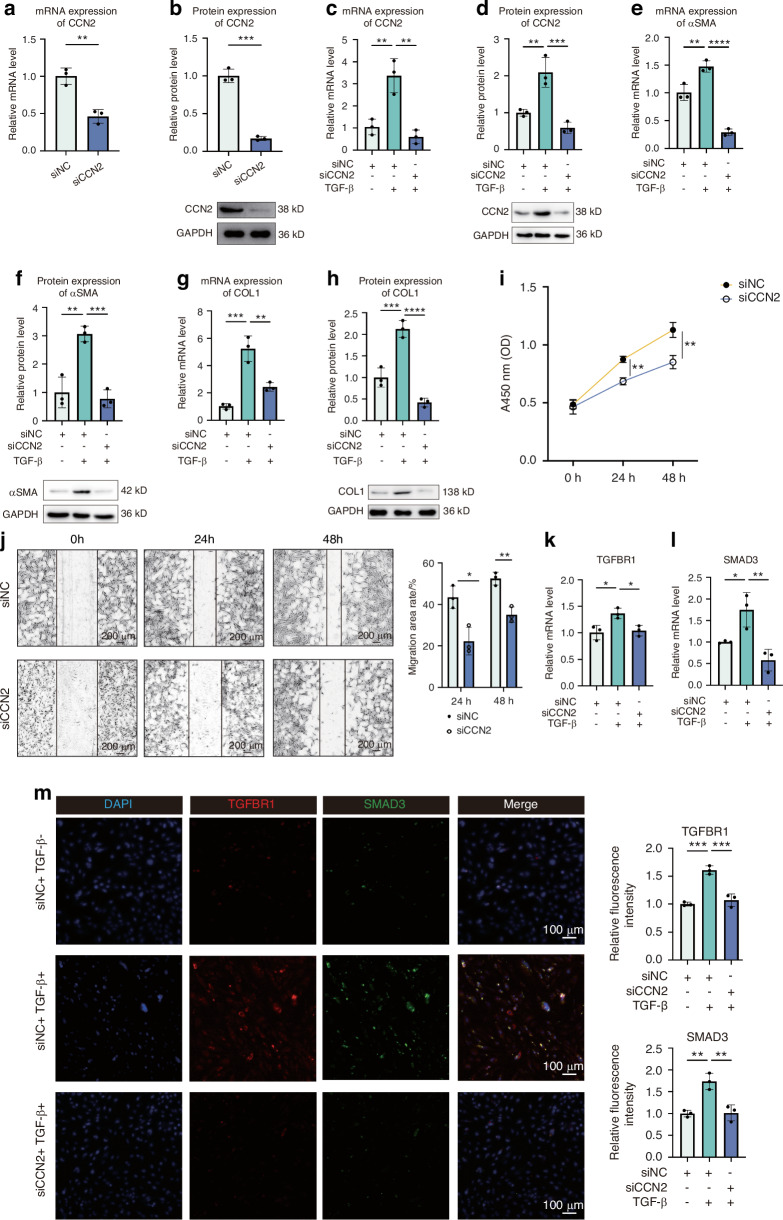
Knockdown of CCN2 attenuated the pro-fibrotic functions of fibroblasts by downregulating TGFBR/SMAD signalling pathway. **a**, **b** Expression of CCN2 with siCCN2 or with siNC (non-target control siRNA) assessed by RT-qPCR and western blotting (*n* = 3). **c**–**h** Expression of CCN2 and other activation markers (αSMA and COL1) after culturing with TGF-β (4 ng/mL) in transfected fibroblasts analyzed by RT-qPCR and western blotting (*n* = 3). **i** Proliferations of the transfected cells measured using the CCK-8 assay at different time intervals (*n* = 3). **j** Cell migration evaluated by wound-healing assays (*n* = 3). Representative images (original magnification, ×40) are shown on the left. Bar = 200 μm. The migration area rates in multiple time intervals are presented on the right. **k**, **l** Expression of TGFBR1 and SMAD3 after culturing with TGF-β (4 ng/mL) in transfected fibroblasts analyzed by RT-qPCR (*n* = 3). **m** Left: Representative immunofluorescence images of TGFBR1 and SMAD3 in transfected fibroblasts after TGF-β (4 ng/mL) stimulation. Right: Bar plots showing relative fluorescence intensity of TGFBR1 and SMAD3. OD, optical density; *P < 0.05; ** *P* < 0.01; *** *P* < 0.001; **** *P* < 0.000 1. Data are shown as mean ± SD

To examine the functions of CCN2 in fibroblasts, CCN2 expression was suppressed using small interfering RNA (siRNA). The inhibitory effects of the siRNA are illustrated in Fig. 5a, b. As a target gene of TGF-β, CCN2 expression was found to increase dramatically after TGF-β stimulation in both the RT-qPCR assay and western blotting (Fig. 5c, d), while the TGF-β-induced CCN2 expression was inhibited after siCCN2 transfection (Fig. 5c, d).

Regarding pro-fibrotic functions, RT-qPCR and western blot analysis consistently confirmed that several typical activation markers, including αSMA and COL1, could be induced by TGF-β stimulation (Fig. 5e–h). This TGF-β-induced activation could also be greatly attenuated by siCCN2 transfection (Fig. 5e–h). Additionally, we found that fibroblast proliferation could be strongly suppressed by siCCN2 transfection (24 h, *P* = 0.001; 48 h, *P* = 0.005) (Fig. 5i). Using a wound-healing assay, we also observed robust migration inhibition after 24 h and 48 h incubation (*P* = 0.013; *P* = 0.003) (Fig. 5j).

Previous studies have indicated the role of TGFBR/SMAD signalling pathway in CCN2-induced fibrosis^54–56^ by increasing binding of TGF-β to corresponding receptors.^57,58^ To examine the potential TGFBR and SMAD activation, we performed RT-qPCR and immunofluorescence staining for TGRBR1 and SMAD3 in fibroblasts with siRNA transfection. We found that both mRNA and protein expression of TGFBR1 and SMAD3 were increased under treatment with TGF-β (Fig. 5k–m). After knockdown of CCN2, expression of both markers were decreased (Fig. 5k–m). Thus, we discovered that CCN2 knockdown strongly inhibited the TGF-β-induced activation, proliferation, and migration of fibroblasts by downregulating the TGFBR/SMAD signalling pathway. These data also indicated that CCN2, which is autocrinally produced by fibroblasts, could amplify the activation of the TGF-β pathway.

### The clinical utility of CCN2 expression and knee AF

To confirm whether CCN2 is a potential biomarker in knee AF and whether CCN2 could be induced by TGF-β, we recruited 30 additional patients, with 10 in the AF group and 20 in the NC group. The baseline characteristics, including sex, age, body mass index, and knee-related patient-reported outcome measures, are listed in Table S3.

The synovial tissues were obtained intraoperatively. An immunofluorescent staining of CD68, DCN and αSMA for macrophages, fibroblasts and myofibroblasts respectively were performed.^31,59,60^ A co-localization of these three types of cells were observed in AF group, while such co-localization is less obvious in NC group (Fig. S6).

**Fig. 6:**
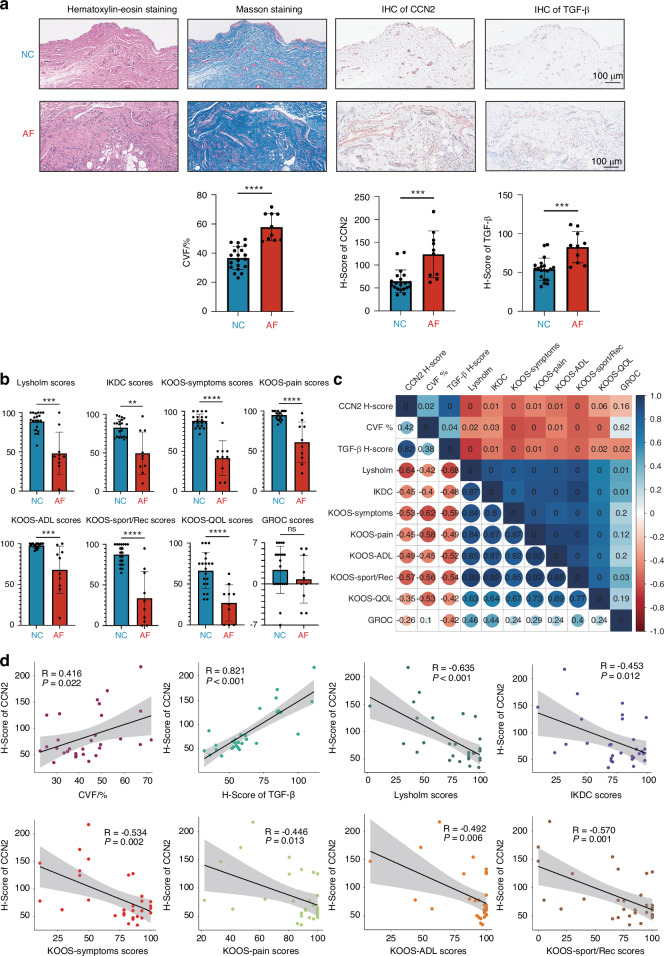
The clinical utility of CCN2 expression and knee AF. **a** Up: Representative images of H&E staining, Masson’s trichrome staining, and immunohistochemistry staining of both CCN2 and TGF-β of synovial tissues in the NC (*n* = 20) and AF (*n* = 10) groups. Bar = 100 μm. Down: Quantification of CVF, H-score of CCN2, and H-score of TGF-β in both groups. **b** Results of multiple knee-related patient-reported outcome measures in both groups showing a significant decrease in the scores in the AF group. **c** Correlation matrix of CVF, H-score of CCN2, H-score of TGF-β, and the knee-related patient-reported outcome measures. The lower part of the matrix showed the correlation coefficient, while the upper part showed the *P*-value of each pair. **d** Linear regression of the H-score of CCN2 and related factors (CVF, H-score of TGF-β, the Lysholm scale, IKDC scale, and KOOS scale). The correlation coefficient (R) and the *P*-value are shown in the images. CVF collagen volume fraction, IKDC International Knee Documentation Committee, KOOS Knee Injury and Osteoarthritis Outcome Score, ADL activities of daily living, Sport/Rec sport and recreation function, QOL knee-related quality of life, GROC Global Rating of Change Score; ** *P* < 0.01; *** *P* < 0.001; **** *P* < 0.000 1; ns, no significance. Data are shown as mean ± SD

Additionally, H&E staining, Masson staining, and immunohistochemistry (IHC) assay for CCN2 and TGF-β were performed with representative histological slides illustrated in Fig. 6a. More connective tissue with more collagen was observed in the AF group (Fig. 6a). We evaluated the collagen volume by collagen volume fraction (CVF) and observed a significantly higher CVF in the AF group than in the NC group (*P* < 0.000 1), indicating a higher volume of collagen deposition (Fig. 6a). The IHC results of CCN2 and TGF-β were also assessed with H-score by two independent, experienced medical pathologists concealed of allocation of samples. Significant differences were observed in the H-score of CCN2 (AF:123.7 ± 48.3; NC:64.6 ± 24.3; *P* < 0.001) and the H-score of TGF-β (AF:82.8 ± 19.3; NC:54.1 ± 14.1; *P* < 0.001) between both groups, demonstrating a higher expression of CCN2 and TGF-β in the AF group (Fig. 6a). We then collected knee-related patient-reported outcome measures for all patients, including the Lysholm scale, International Knee Documentation Committee (IKDC) scale, Knee Injury and Osteoarthritis Outcome Score (KOOS) scale, and Global Rating of Change Score (GROC).^61–63^ Significant differences were observed in all measures except GROC (Fig. 6b), suggesting great distress and loss of knee function in patients with knee AF, consistent with previous studies.^10^

To gain insight into the correlation between the H-score of CCN2 and related factors, Pearson correlation analysis was performed. The H-score of CCN2 was positively correlated with both CVF and the H-score of TGF-β (Fig. 6c). Consistently, the H-score of CCN2 was also found to be negatively correlated with the overall knee function (Lysholm and IKDC), knee-related symptoms (KOOS-Symptoms), knee pain (KOOS-Pain), activities of daily living (KOOS-ADL), and sport and recreation functions (KOOS-Sport/Rec) (Fig. 6c). Linear regression analyses of these factors suggested that the high level of CCN2 could be induced by the high level of TGF-β, leading to excessive collagen production and poor knee function (Fig. 6d).

**Fig. 7:**
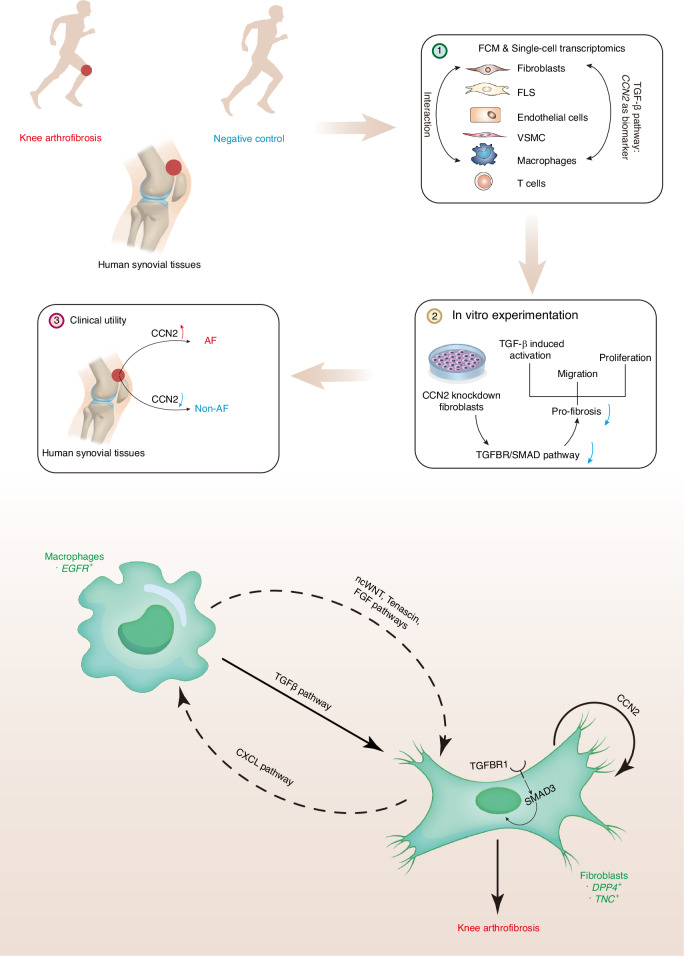
Schematic representation of the molecular mechanism underlying knee arthrofibrosis. Human synovial tissues from patients with knee arthrofibrosis (AF) and negative controls (NC) were obtained. First, with the FCM and single-cell transcriptomic analysis, we identified fibroblasts, macrophages, FLS, endothelial cells, VSMC, and T cells as main components of synovial tissues. The fibroblast-macrophage interactions were investigated, and *CCN2* in the TGF-β pathway was identified as a potential biomarker for knee AF. Second, in vitro experimentations revealed that autocrine of CCN2 is essential in the pro-fibrotic functions (activation, proliferation, and migration) of fibroblasts through TGFBR/SMAD pathway. Then, we verified CCN2 expression in clinical patients and discovered that TGF-β induced CCN2 is positively related to AF. Schematic molecular mechanism underlying the knee arthrofibrosis is shown at the bottom. Macrophages initiate the fibrotic casade by activating multiple pro-fibrotic pathways, including TGF-β signaling pathways and others (the ncWnt, tenascin, and FGF signaling pathways). The fibroblasts, as receivers, affect macrophages via the CXCL signaling pathway. Additionally, autocrine of CCN2 by fibroblasts promoted fibrosis through TGFBR/SMAD pathway, thus leading to the knee arthrofibrosis

Consistently, a strong correlation was observed between the expression of CCN2 and TGF-β, collagen production, and poor outcome of patients. These results were consistent with the findings in our scRNA-seq analysis and in vitro experiments, suggesting both TGF-β pathway and TGF-β induced-CCN2 might be involved in regulating the pathogenesis of knee AF (Fig. 7).

## Discussion

Many efforts have been made to investigate knee AF; however, the molecular mechanisms and cellular landscape of knee AF remain unclear. Therefore, we generated single-cell transcriptomes of knee AF (AF group) and negative control (NC group) synovial tissues to reveal the cellular characteristics and underlying mechanisms of knee AF.

To our knowledge, this is the first comprehensive study to perform single-cell transcriptomic analysis of human synovial tissues in knee AF following ACL reconstruction. Our results indicated six cell types in the synovial tissue post-reconstruction: fibroblasts, macrophages, FLS, VSMC, endothelial cells, and T cells. Consistent with previous studies, in our study, fibroblasts and macrophages significantly increased in the AF group.^13^ The fibroblast-macrophage interaction have been well described in various fibrotic diseases. For example, macrophages can regulate fibroblasts by secreting multiple cytokines.^49^ However, the cell composition and the interaction between both cell types in knee AF remain unknown.^39,48^ In this study, fibroblasts were subclassified into three groups based on their distinct functions, and three subpopulations of macrophages were also identified. The change in abundance of macrophage subpopulation between both groups was supported by previous researches.^64^ Specific pro-fibrotic subpopulations of fibroblasts (Fib1 and Fib2) and macrophages (Macro3) were then proposed. By CellChat analysis, we observed the fibroblast-macrophage interaction through pathways, including ncWNT, TGF-β, tenascin, FGF, and CXCL. Furthermore, the TGF-β pathway-related gene, *CCN2*, of fibroblasts were suggested to be a potential biomarker for knee AF. These data highlight the importance of *CCN2* in knee AF.

Regarding the potential function of CCN2, multiple studies have shown that CCN2 serves as a central mediator of persistent fibrosis which amplifies TGF-β-induced activation and effective inhibition of CCN2 can potentially reverse the process of persistent fibrosis, including systemic sclerosis, amyotrophic lateral sclerosis, renal fibrosis and pulmonary fibrosis.^58,65–70^ In this study, we confirmed the induction of CCN2 by TGF-β stimulation by in vitro experiments and discovered that by knocking down CCN2, pro-fibrotic functions (activation, cell proliferation and migration) in response to TGF-β stimulation could be significantly inhibited mainly due to the downregulation of TGFBR/SMAD signaling pathway. These findings added another route for knee AF, the “autocrine fibroblast circuit”, in which TGF-β induces CCN2 and CCN2 in turn amplifies the TGF-β functions.^49^ In another cohort of patients, we observed a co-localization of fibroblasts, myofibroblasts and macrophages in AF group indicating a possible cell interaction among these cells. Additionally, we found the H-score of CCN2 was positively correlated with CVF and the H-score of TGF-β but negatively associated with multiple knee-related patient-reported outcome measures (Lysholm, IKDC, KOOS-Symptoms, KOOS-Pain, KOOS-ADL, KOOS-Sport/Rec). These clinical results also suggest that over-expressed TGF-β would lead to excessive CCN2 which may be vital in fibrosis leading to the poor outcome of patients. Thus, considering disease specificity, CCN2 could serve as a fine auxiliary biomarker for prognosis. Additionally, with robust clinical trials and drugs (FG-3019, pravastatin, etc.) targeting on CCN2, CCN2 may also serve as a promising therapeutic target for knee AF with high precision.^54^

There are several limitations in this study: 1) The number of patients enrolled in this study is limited and though we have matched age, sex and BMI, other factors including smoking and diabetes status were not matched. Thus, a larger longitudinal cohort of patients with well-designed matching is needed. 2) We mainly focused on CCN2 in this study, and more factors and biological interaction mechanisms still need to be addressed in future work.

Taken together, our single-cell transcriptome atlas of human knee AF reveals both fibroblast-macrophage interaction of TGF-β pathway and an autocrine fibroblast circuit of CCN2 in knee AF, suggesting the utility of CCN2 as a novel biomarker.

## Materials and methods

### Ethics

The local ethics committee of the Shanghai Sixth People’s Hospital Affiliated to Shanghai Jiao Tong University School of Medicine approved all human tissue protocols (2022-KY-016(K)). All patients provided informed consents and the study was performed in accordance with the Declaration of Helsinki.

### Acquiring human patient samples

For the “Biomarker Identification” part, patients with ACL injury and a loss of range of motion were treated properly in the ACL reconstruction surgery. After the surgery, patients who underwent a year of rehabilitation and determined to remove the internal fixation by another arthroscopic surgery were recruited. Those with knee arthrofibrosis were allocated in the AF group (arthrofibrosis; *n* = 3) while others were allocated in the NC group (negative controls; *n* = 3). The diagnosis of knee arthrofibrosis was based on the range of motion (ROM) using a goniometer and medical records while blood tests and MRI were used to rule out infection or other specific causes.^13^ Additionally, the diagnosis of ACL tears was based on MRI and physical examination (the Lachman test and the Anterior Drawer test) and the ACL reconstruction was performed after the six weeks of “acute phase” to reduce inflammation.^71–73^ Patients in both groups were matched according to gender, age and BMI. For the “Biomarker Validation” part, similar procedures were carried out again and 30 patients were recruited with 10 in AF group and 20 in NC group. All surgeries above were performed by JZ, the senior sports medicine surgeon of the Department of Sports Medicine of the Shanghai Sixth People’s Hospital Affiliated to Shanghai Jiao Tong University School of Medicine. Synovial tissue was collected from the supra-patella pouch as described in previous studies, since it can serve as a good representative of the fibrosis knee and is technically more practical.^17^ The tissue was identified as synovium by characteristic features: a fibrous tissue, pink or greyish in appearance.^27^ Additionally, knee-related patient-reported outcome measures for all patients, including the Lysholm scale, International Knee Documentation Committee (IKDC) scale, Knee Injury and Osteoarthritis Outcome Score (KOOS) scale, and Global Rating of Change Score (GROC).^61–63^

### Processing of human tissue

Tissue excised was transported from the operating room to the laboratory in ice-cold tissue storage solution (#21903-10, SHBIO). When reaching the laboratory, tissues were transported into several sterile culture dishes with 10 mL 1x Dulbecco’s Phosphate-Buffered Saline (#14287080, Thermo Fisher) on ice to remove the residual tissue storage solution. These tissues were then cut into smaller pieces (~ 1–2 mm^3^) by forceps and surgical scissors and then transferred to a C-tube (#130-093-237, Miltenyi) in a digestion solution dissolved in PBS with 0.25% Trypsin (#25200-072, Thermo Fisher), 10 μg/mL DNase I (#11284932001, Sigma) 5% Fetal Bovine Serum (#SV30087.02, Thermo Fisher). Mechanical disruption of samples was performed on a gentle MACS dissociator system (Miltenyi) with the program m_Spleen 04.01.^27^ The samples were digested with a shaking speed of 50 r/min for about 20 min. After incubation, the suspension was passed through a 100-μm cells strainer (#CSS013100, Jet-Biofiltration) following by a 40-μm cells strainer (#CSS013040, Jet-Biofiltration) and was centrifuged for 10 min at 800 *g* at 4 °C. After removal of the remaining red blood cells in samples by RBC lysis buffer (#00-4333-57, Thermo Fisher) with an incubation time of 1 min, the suspension was then centrifuged again for 5 min at 500 *g* at 4 °C. The single cells were washed with 1x DPBS containing 2% FBS and were stained with 0.4% Trypan blue (#T10282, Thermo Fisher) to check the viability on Countess® II Automated Cell Counter (#AMQAX1000, Thermo Fisher).

### Flow cytometry cell analysis

Synovial cell suspensions were stained with an 18-color flow cytometry panel to identify synovial stromal cell types and leukocytes. Antibodies included anti-CD3-BB700 (#552852, BD Pharmingen, RRID: AB_2869885), anti-CD19-BWV395 (#557921, BD Pharmingen, RRID: AB_2738272), anti-CD45-BV510 (#557833, BD Pharmingen, RRID: AB_2738067), anti-CD14-FITC (#555397, BD Pharmingen, RRID: AB_395798), anti-CD31-PE (#555446, BD Pharmingen, RRID: AB_395839) and anti-PDPN-BV42 (#566456, BD Pharmingen, RRID: AB_2739735). Since PDPN is expressed primarily in synovial fibroblasts, we chose PDPN as a marker of synovial fibroblasts.^27,74^ Isolated cells were resuspended in 1% FBS/PBS on ice to reach a final concentration of 1 × 10^6^ cells per mL. Cells were then incubated with Fc-Block (#564219, BD Pharmingen) for 10 min and then incubated with the above antibodies for 30 min on ice protected from light. Single cells were then washed 1 mL 1% FBS/PBS. Data analysis was performed using FlowJo.

### 10X Single cell RNA-seq library construction and sequencing

After acquiring the synovial cell suspensions, beads with unique molecular identifier (UMI) and cell barcodes were loaded close to saturation to ensure that each synovial cell was paired with a bead in a Gel Beads-in-emulsion (GEM). After cell lysis, polyadenylated RNA molecules were hybridized to the beads. Then the beads were retrieved into a single tube for reverse transcription. During the synthesis of cDNA, each cDNA molecule was tagged with UMI and cell labels on the 5’end (that is, the 3’end of a messenger RNA transcript), indicating its cell origin. Briefly, 10× beads then went through procedures including second-strand cDNA synthesis, adaptor ligation, and universal amplification. Furthermore, sequencing libraries were prepared by using randomly interrupted whole-transcriptome amplification products to enrich the 3’ end of the transcripts linked with the cell barcode and UMI. The remaining procedures, including the library construction, were performed according to the standard manufacturer’s protocol (CG000206 Rev D). Sequencing libraries were quantified by utilizing the High Sensitivity DNA Chip (#2100 Bioanalyzer, Agilent) on a Bioanalyzer 2100 and the Qubit High Sensitivity DNA Assay (Qubit 4.0 Flourometer, Thermo Fisher Scientific). The libraries were finally sequenced on NovaSeq6000 (Illumina) with 2 × 150 chemistry.

### Single cell RNA sequencing data pre-processing and quality control

The raw sequencing reads were processed using the Cell Ranger 7.1.0 pipeline with default and recommended parameters. FASTQs generated from Illumina sequencing output were aligned to the human genome, version GRCh38-2020-A, using the STAR algorithm. Gene-Barcode matrices were then generated for each individual sample by counting UMIs and filtering non-cell associated barcodes. Finally, we generated a gene-barcode matrix containing the barcoded cells and gene expression counts. Further process, analysis and visualization was performed with the use of Scanpy^75^ toolkit (v1.9.1) based on Python software (v3.10). Quality control was performed with thresholds for the following metrics: (i) detected genes per cell ranging from 200 ~ 6 000, (ii) maximum fraction of mitochondrial reads below 10%, and (iii) number of mRNAs per cell below 23 000. Cells that did not meet these criteria were filtered.

### Data integration, dimension reduction and cell clustering

Total-count normalize the data matrix to 10 000 reads per cell by using the function scanpy.pp.normalize_total, so that counts become comparable among cells. Then the data was logarithmized through the function scanpy.pp.log1p and the top 3 000 most highly-variable genes were identified by the scanpy.pp.highly_variable_genes function of Scanpy. We also scaled each gene to unit variance using the function scanpy.pp.scale with parameter max_value = 1. After data preprocessing, we performed dimensional reduction of the data by running principal components analysis (PCA). A PCA matrix was calculated to reveal the main axes of variation and denoise the data through the function scanpy.tl.pca with parameter svd_solver=’arpack’, n_pcs=6. By using the PCA representation of the data matrix, we computed the neighborhood graph of cells and presented it in two dimensions with Uniform Manifold Approximation and Projection (UMAP) where we set the min_dist as 0.7 for better visualization. In the UMAP representation of the data, no significant batch effect was found so we here did not have the procedures of correct the batch effect. The clustering analysis was performed via the Leiden graph-clustering method (community detection based on optimizing modularity) with resolution of 0.1. For sub-clustering analysis, we applied a similar procedure, including the variable genes identification, dimension reduction and clustering analysis.

### Annotating cell clusters

We identified the differentiation marker genes in the specific cluster compared to remaining clusters through the function scanpy.tl.rank_ genes_groups with the Wilcoxon Rank Sum Test. The annotation of cell clusters was based primarily on the automatic annotation (the SCSA) while annotation of tissue-specific cells (fibroblast-like synoviocytes and fibroblasts) was based on The Human Protein Atlas (https://www.proteinatlas.org), CellMarker (http://xteam.xbio.top/CellMarker) and published literatures.^76^ Specific markers of cells were listed as follows: fibroblast-like synoviocytes (THY1- Clic5^+^ Has1 ^+^ ),^24^ fibroblast (THY1 ^+^ LUM ^+^ ),^24,25^ macrophages (CD14 ^+^ MS4A4A ^+^ ),^26,27^ endothelial cells (EMCN ^+^ VWF ^+^ ),^77^ vascular smooth muscle cells (Notch3 ^+^ ACTA2 ^+^ )^24^ and T cells (CD2 ^+^ CD3D ^+^ ).^78^

### Enrichment analysis

Gene Ontology (GO) analysis is a computational method used in bioinformatics to understand the biological functions of genes and their products, widely used in high-throughput experiments.^78,79^ The enrichment analysis of GO in the cell clusters were performed by using the Metascape database (v3.5.20230101) to determine if there is a significant overrepresentation of certain GO terms among the genes of interest.^80^ Genes with *P* < 0.01 were selected and submitted to the Metascape database and GO terms with *P* < 0.05 were selected and terms of interest were demonstrated in heatmaps.

### RNA velocity

The RNA velocity analysis was based on the relative abundance of unsoliced and spliced mRNA to estimate the rates of gene splicing and degradation as described previously.^37^ RNA velocity analysis was conducted by using velocyto v.0.17.17 and scVelo v.0.2.5 which generalized RNA velocity to transient cell states through dynamical modeling. In particular, to count spliced and unspliced reads for each sample, the 10X velocyto pipeline was run in the filtered cell ranger generated BAM files, while for single-cell RNA velocity inference, the dynamical model of scVelo was applied. This method allowed us to make use of transient cellular states to predict the progressions of transcriptomic signatures of cells along the identified trajectories. For RNA velocity analysis of cells in this research, we used the UMAP calculated in clustering for visualization through the function scvelo.pl.velocity_embedding_stream with pararmeter density=3, smooth=1.

### CellChat analysis

Potential cell-cell interactions were investigated between all fibroblast subpopulations and macrophages subpopulations. We performed the CellChat (1.6.1) platform developed by Jin, Guerrero-Juarez et al.^47^ Default parameterizations were used throughout, and Secreted Signaling, ECM-Receptor, and Cell-Cell Contact relationships were considered.

### PPI network integration

Protein-protein interaction (PPI) of the top 50 DEGs of fibroblasts between AF and NC groups were constructed by Search Tool for the Retrieval of Interacting Genes database (STRING, https://string-db.org/).^81^ Clustering of PPI network was performed by kmeans clustering.

### Cell preparation

The human fibroblast cell line (#AW-CELLS-211, HSF-SV40T) used in this study were obtained from AnWei-sci. Fibroblasts were cultured in Dulbecco’s modified Eagle’s medium (#C11995500BT, Gibco) containing 10% fetal bovine serum (FBSSR-01021-500, OriCell) at 5% CO_2_ and 37 °C. Fibroblasts from passages 4–6, which were a homogeneous cell population, were used in our experiments.

### Transfection of siRNAs

The CCN2 siRNAs and control siRNAs were obtained from Genomeditech. The target sequences of CCN2 siRNA are listed as follows: GCUAAAUUCUGUGGAGUAUTT, AUACUCCACAGAAUUUAGCTT. Fibroblasts at 60%-70% confluence were transfected with the above siRNAs or the corresponding NC using Lipofectamine 3000 (#L3000001, Thermo Fisher Scientific) according to the manufacturer’s protocol. Experiments were performed 48–72 h after transfection. Cells were incubated for a further 24 h with the addition of TGF-β1 (#HZ-1011, Proteintech) with a concentration of 4 ng/mL before being lysed for Western blot analysis and RT-qPCR analysis.

### RNA isolation and quantitative RT–qPCR

RNA was extracted with the EZ-press RNA Purification Kit (#B0004D, EZBioscience) according to the manufacturer’s instructions. RT–qPCR was carried out with ROX2 plus (#EZB-qRT-R2, EZBioscience) and analyzed with the standard procedures. All primers used in our study are listed as follows: CCN2 (Forward-5′- GAAATGCTGCGAGGAGTG- 3′; Reverse-5′- CCCACAGGTCTTGGAACA -3′), αSMA (Forward-5′- GTTACGAGTTGCCTGATGG -3′; Reverse-5′- AGGTGGTTTCATGGATGC -3′), COL1 (Forward-5′- TCTGAAACCCTCCTCCACA -3′;Reverse-5′- GCTTCCTCCCACCCCTA -3′),TGFBR1 (Forward-5′-TGATAAAACTTGCTCTGTCCA -3′; Reverse-5′- ACCAACAATCTCCATGTGAA -3′), SMAD3 (Forward-5′- TTCAACAACCAGGAGTTCG -3′; Reverse-5′- TGCACATTCGGGTCAAC -3′), GAPDH (Forward-5′- CAGGAGGCATTGCTGATGAT -3′; Reverse-5′- GAAGGCTGGGGCTCATTT -3′). Target gene expression relative to glyceraldehyde-3-phosphate dehydrogenase (GAPDH) expression was calculated by the 2^-ΔΔCt^ method.

### Western blot analysis

Fibroblasts were lysed with RIPA buffer containing protease inhibitors and phosphatase inhibitors, and the protein concentrations were detected with a BCA protein assay kit (#P0011, Beyotime). Equal amounts of protein were loaded onto 10% polyacrylamide gels and then transferred onto polyvinylidene difluoride membranes. The membranes were blocked with TBS/Tween-20 containing 5% nonfat milk for 1 h. Then, the membranes were incubated with the primary antibodies anti-CTGF (#ab209780, Abcam, 1:1 000), anti-αSMA (#ab124964, Abcam, 1:1 000), anti-COL1 (#14695-1-AP, Proteintech, RRID: AB_2082037, 1:1 000) and anti-GAPDH antibody (#A20120A0701, Biotnt, 1:1 000) at 4 °C overnight. The membranes were then incubated with anti-rabbit IgG (A20120A0704, Jackson ImmunoResearch, RRID: AB_2337913) for 1 h at room temperature, and were visualized according to standard protocols. Signals from the bands were quantified by Image J software (v 2.0.0).

### Wounding migration assay

Fibroblasts were seeded into 6-well plates (#0415B, NEST Biotechnology) and transfected with CCN2 siRNA or corresponding NC as described above. Then, the cells were scratched with 200 μL pipette tips and treated with DMEM containing 10% FBS for 24 h to 48 h. Migration area was quantified by subtracting the blank area at 24 h and 48 h from the blank area at 0 h by using Image J software (v 2.0.0), and subsequently a migration area rate was calculated.

### Cell proliferation assay

Cell proliferation assays were conducted using the Cell Counting Kit-8 (CCK-8) (#C0039, Beyotime) according to the supplier’s instructions. 100 μL of cell suspension containing 5 × 10^3^ cells was added onto each well of a 96-well plate. After transfection with CCN2 siRNA or corresponding NC, 10 μL of CCK-8 reagent was added to each well of the 96-well plate and the cells were incubated for 1 h at the 0, 24, 48 h time points. Absorbance was then read at a wavelength of 450 nm by using a microplate reader (#Multiskan FC, Thermo Fisher Scientific).

### Immunofluorescent cell staining

For immunofluorescent cell staining, HSF cells were seeded on collagen-coated coverslips. After transfection and TGF-β stimulation, cells were fixed with 4% paraformaldehyde (PFA), permeabilized with 0.2% Triton X-100, blocked, and then incubated with primary antibodies for SMAD3 (#AF6362, Affinity, RRID: AB_2835210, 1:200) and TGFBR1 (#BF8256, Affinity, 1:200) overnight. Cells were then incubated with second antibodies (#AS026, ABclonal, 1:1 000; #AS011, ABclonal, 1:1 000). DAPI was used for staining for the nuclei. Images of the stained sections were digitized and analyzed using a microscope (#DM6B, Leica).

### Hematoxylin-Eosin (HE) staining

Synovial tissues were dehydrated in graded alcohol and then embedded in paraffin and a series of 4.0 μm sections were cut. HE staining was then executed according to the standard procedures. The tissue sections were transparent with xylene and then mounted with neutral balsam.

### Masson staining

Masson staining was carried out according to the standard procedures to assess the level of collagen deposition. The collagen volume fraction (CVF) was calculated as the blue area that indicated collagen divided by the total synovial tissue area under direct vision by 2 experienced pathologists independently. Therefore, the higher the CVF the more intense was the fibrosis. Image J software (v 2.0.0) was used for calculation.

### CCN2/TGF-β immunohistochemistry staining

CCN2/TGF-β immunohistochemistry staining was performed on all tissue sections. CCN2 was stained with anti-CCN2 (#TA806803, Origene, 1:50) antibody and TGF-β was stained with anti-TGF-β (#21898-1-AP, Proteintech, RRID: AB_2811115, 1:800) antibody according to the standard procedures of immunohistochemistry. The intensity and percentage of CCN2/TGF-β expression in cells were evaluated by two independent, experienced pathologists independently with Image J software (v 2.0.0) who are concealed of allocation of samples. H-score was adopted as described preciously to evaluate the staining results: adding the results of multiplication of the percentage of cells with staining intensity ordinal value (scored from 0 for “no signal” to 3 for “strong signal”).^82,83^

### Immunofluorescence staining

Tissue sections were blocked with 3% H_2_O_2_ for 30 minutes at room temperature and then covered with 10% goat serum and incubated for 30 minutes at 37 °C. DCN, CD68 and αSMA was stained by anti-DCN (#14667-1-AP, Proteintech, 1: 2 000), anti-CD68 (#ab201340, Abcam, 1:8 000) and anti-αSMA (#67735-1-Ig, Proteintech, 1:5 000) respectively. A standard TSA staining protocol was then carried out with iFluor® 488 tyramide (#11060, 45100, AAT Bioquest). After washing, sections were mounted with fluoromount and coated with coverslips. Images were observed with a Leica microscopy.

### Statistics

Statistical analysis of scRNA-seq were described above. Representative continuous variables were expressed as mean ± SD, and categorical variables were represented as *n* (%). In vitro experiments in this study were independently repeated at least 3 times. To reduce baseline variability between independent experiments, the quantitative analyses of western blot analysis and mRNA expression were normalized based on the mean of the control. The normality was checked on the raw data for continuous variables. If the data meet the criteria of normal distribution, two-tailed student’s *t*-test or one-way analysis of variance (ANOVA) were used for two groups and multiple comparisons, respectively. If the data do not meet the criteria of normal distribution, Mann-Whitney test or Kruskal-Wallis test was performed for two groups and multiple comparisons, respectively. The Chi-squared test was used for categorical variables. For groups of ≥ 3, proper post hoc test was applied for calculating statistical significance. Pearson correlation analysis and the linear regression were performed for H-score of CCN2 and other relevant factors. A *P*-value < 0.05 was considered significant. Statistical analyses were performed with SPSS (v 26.0.0.0) and R (v 4.1.3).

## Supplementary information

Supplementary file

## Data Availability

Raw and processed single-cell RNA sequencing dataset have been deposited in the the National Center for Biotechnology Information (NCBI) with the accession code PRJNA1014406. The data that support the findings of this study are available from the corresponding author upon reasonable request.

## Code availability

Scripts for processing the single-cell data, RNA velocity analysis, Enrichment analysis, cell-cell interaction analysis and visualization are available on GitHub (https://github.com/CCCKW/Knee-Arthrofibrosis/tree/main).
