# Supplementary material for: CCN2 mediates fibroblast-macrophage interaction in knee arthrofibrosis based on single-cell RNA-seq analysis
Source: Bone Res. 2025 Feb 24;13:26. doi: 10.1038/s41413-025-00400-9 (PMC11850813; doi:10.1038/s41413-025-00400-9)
Supplement: Supplementary file 1 — Supplementary file [file 41413_2025_400_MOESM1_ESM.docx]

**Supplementary Tables and Figures**

Table supplement 1. Characteristic of baseline information of enrolled patients in the “Biomarker Identification” part

| Patients | AF-1 | NC-1 | AF-2 | NC-2 | AF-3 | NC-3 |
| --- | --- | --- | --- | --- | --- | --- |
| Gender | Female | Female | Male | Male | Male | Male |
| Age (years) | 26 | 46 | 32 | 25 | 28 | 27 |
| Height (m) | 1.65 | 1.68 | 1.76 | 1.82 | 1.91 | 1.72 |
| Weight (kg) | 53 | 67 | 65 | 92 | 100 | 64 |
| BMI (kg/m^2^) | 19.5 | 23.7 | 21.0 | 27.8 | 27.4 | 21.6 |
| Affection Side | Left | Right | Left | Right | Left | Left |
| Level of knee arthrofibrosis | Mild | / | Mild | / | Mild | / |
| Score of Lysholm Scale | 84 | 74 | 94 | 100 | 97 | 70 |
| Score of IKDC Scale | 66 | 61 | 86 | 83 | 63 | 71 |
| Score of KOOS-Symptoms | 67.86 | 67.86 | 82.14 | 92.86 | 60.71 | 71.43 |
| Score of KOOS-Pain | 83.33 | 94.44 | 97.22 | 100.00 | 94.44 | 83.33 |
| Score of KOOS- ADL | 95.59 | 91.18 | 100.00 | 100.00 | 95.59 | 88.24 |
| Score of KOOS-Sport/Rec | 70.00 | 70.00 | 100.00 | 100.00 | 70.00 | 75.00 |
| Score of KOOS-QOL | 50.00 | 50.00 | 75.00 | 93.75 | 43.75 | 37.50 |
| Score of GROC | 0 | 5 | -1 | 0 | 0 | 6 |

BMI, Body Mass Index; IKDC, International Knee Documentation Committee; KOOS, Knee Injury and Osteoarthritis Outcome Score; ADL, Activities of daily living; Sport/Rec, Sport and recreation function; QOL, knee-related quality of life; GROC, Global Rating of Change Score.

Table supplement 2. Top 50 DEGs of fibroblasts in AF compared with NC

|  | Genes | *P*-Value | logFC |
| --- | --- | --- | --- |
| 1 | CCN2 | 0 | 1.0244951 |
| 2 | SLC35F1 | 0 | 2.1910052 |
| 3 | FMOD | 0 | 1.1972795 |
| 4 | ASPN | 0 | 0.61228436 |
| 5 | AQP1 | 0 | 1.0082852 |
| 6 | GALNT15 | 0 | 0.9878066 |
| 7 | MT1E | 0 | 0.8232152 |
| 8 | NDUFA4L2 | 0 | 1.3038507 |
| 9 | KCNMA1 | 0 | 1.1812646 |
| 10 | COMP | 0.00000000000000E+00 | 1.0817099 |
| 11 | CDH13 | 2.75646135790502E-284 | 1.1985133 |
| 12 | LDLRAD4 | 4.30053050167715E-280 | 0.9529436 |
| 13 | COL8A1 | 1.31678200290233E-273 | 1.0302371 |
| 14 | PRELP | 6.03055176819646E-258 | 0.65071887 |
| 15 | SERPINE1 | 4.69237410415373E-252 | 1.5423149 |
| 16 | RHOB | 8.97067339512027E-248 | 0.82176006 |
| 17 | TGFBI | 1.70726377014157E-243 | 1.2141886 |
| 18 | CAPS | 7.08207378155527E-241 | 1.4740516 |
| 19 | NFKBIZ | 1.04225998238207E-232 | 1.1562047 |
| 20 | CRTAC1 | 2.02603564371608E-226 | 0.851332 |
| 21 | CAPG | 2.04957211898411E-206 | 0.98700964 |
| 22 | CYP1B1 | 7.2014754667372E-203 | 0.76378435 |
| 23 | HIVEP2 | 1.69963005323807E-194 | 0.73855144 |
| 24 | HMCN2 | 3.2125379822856E-177 | 0.806349 |
| 25 | PDE4D | 7.92212095028829E-176 | 1.0406373 |
| 26 | HMOX1 | 6.5500578333158E-173 | 1.5144467 |
| 27 | LBH | 3.04523407599959E-171 | 0.98894334 |
| 28 | RFX2 | 3.84931705161736E-157 | 0.8961168 |
| 29 | CASC15 | 2.02659554163708E-156 | 1.1130702 |
| 30 | COLGALT2 | 1.52356403311645E-154 | 0.89323324 |
| 31 | TSPAN2 | 4.28085503858718E-153 | 1.8344785 |
| 32 | MT1X | 1.38508693190498E-152 | 0.63901234 |
| 33 | SLN | 6.50627592424587E-147 | 1.2008153 |
| 34 | CCL2 | 1.58247226687395E-145 | 1.21307 |
| 35 | ADAMTS6 | 3.12006604208879E-138 | 1.0945659 |
| 36 | AGAP1 | 1.69237920897312E-137 | 0.61015165 |
| 37 | PDE10A | 1.74989281314485E-137 | 1.2541842 |
| 38 | CRLF1 | 4.27142131352806E-136 | 0.66067994 |
| 39 | FOXO1 | 1.41902634049442E-135 | 0.6224663 |
| 40 | CADM1 | 5.20232535498895E-132 | 0.69037426 |
| 41 | UGP2 | 2.08812797338534E-129 | 0.5293226 |
| 42 | FIBIN | 1.33072675804317E-128 | 0.9898811 |
| 43 | SAMD4A | 4.21684406671166E-126 | 0.767922 |
| 44 | EZR | 1.93539552343763E-124 | 0.83097327 |
| 45 | NFATC2 | 4.71014425014624E-122 | 0.9846226 |
| 46 | LSAMP | 1.66971204722946E-121 | 1.0969571 |
| 47 | GALNT18 | 7.25477326767083E-121 | 1.3359553 |
| 48 | MT1M | 5.11580566324216E-117 | 0.60275555 |
| 49 | CADM3 | 5.48506072852995E-117 | 0.8346788 |
| 50 | SCG2 | 1.79254331448395E-115 | 0.90072757 |

DEGs, Differentially Expressed Genes; logFC, logFoldChange.

Table supplement 3. Characteristic of baseline information of enrolled patients in the “Biomarker Verification” part

| Parameters | | NC Group | AF Group | *P*-Value |
| --- | --- | --- | --- | --- |
| Number of Patient | | 20 | 10 | *-* |
| Gender | Male | 12 (60%) | 6 (60%) | 1 |
|  | Female | 8 (40%) | 4 (40%) |  |
| Age (years) | | 27.4 ± 8.4 | 35.1 ± 7.4 | 0.035 |
| BMI (kg/m^2^) | | 22.5 ± 4.1 | 23.9 ± 1.8 | 0.229 |
| Affection Side | Left | 11 (55%) | 6 (60%) | 0.794 |
|  | Right | 9 (45%) | 4 (40%) |  |
| Lack of ROM before ACLR | | 20.0 ± 7.8 | 19.1 ± 6.3 | 0.579 |
| Lack of ROM after ACLR | | 0 | 18.6 ± 9.0 | ＜0.0001 |
| Score of Lysholm Scale | | 88.8 ± 11.5 | 48.0 ± 25.8 | ＜0.001 |
| Score of IKDC Scale | | 83.0 ± 10.8 | 49.5 ± 26.2 | 0.003 |
| Score of KOOS-Symptoms | | 88.2 ± 8.1 | 41.4 ± 21.4 | ＜0.0001 |
| Score of KOOS-Pain | | 95.0 ± 5.3 | 61.1 ± 23.9 | ＜0.0001 |
| Score of KOOS-ADL | | 97.7 ± 2.7 | 67.9 ± 27.5 | ＜0.001 |
| Score of KOOS-Sport/Rec | | 87.8 ± 11.8 | 33.5 ± 31.5 | ＜0.0001 |
| Score of KOOS-QOL | | 66.3 ± 21.6 | 26.9 ± 20.9 | ＜0.0001 |
| Score of GROC | | 2.4 ± 3.9 | 0.8 ± 3.9 | 0.373 |

BMI, Body Mass Index; ACLR, Anterior Cruciate Ligament Reconstruction；IKDC, International Knee Documentation Committee; KOOS, Knee Injury and Osteoarthritis Outcome Score; KOOS-ADL: ADL, Activities of daily living; Sport/Rec, Sport and recreation function; QOL, knee-related quality of life; GROC, Global Rating of Change Score.


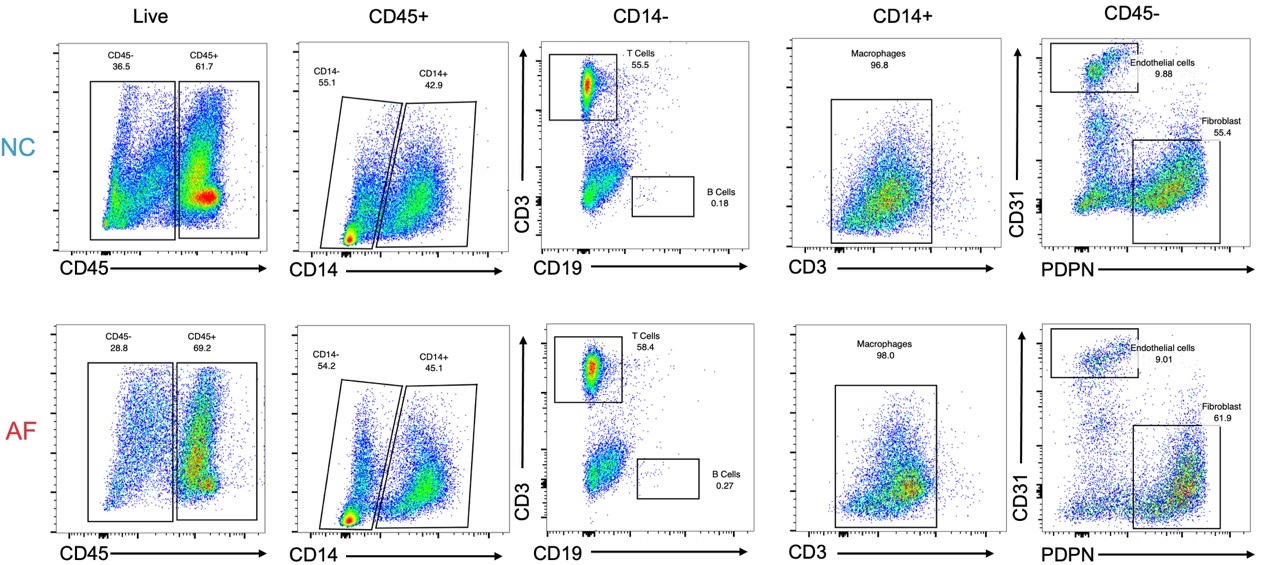


Figure supplement 1. Flow cytometry analysis of cells dissociated from synovial tissue.


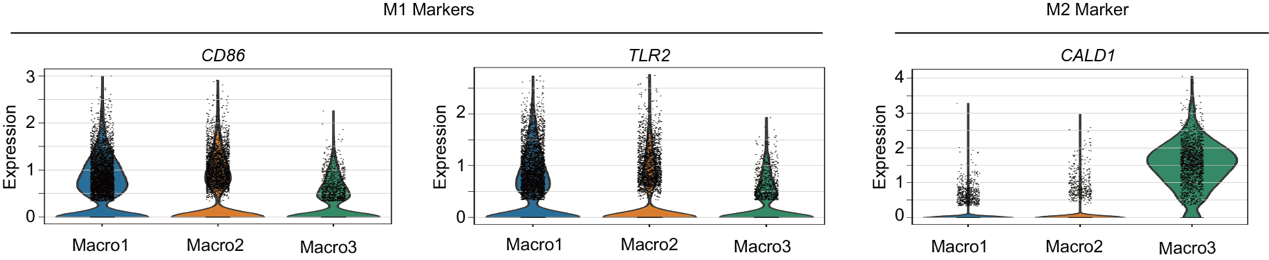


Figure supplement 2. Violin plots showing M1 markers and M2 markers in all subpopulations of macrophages.


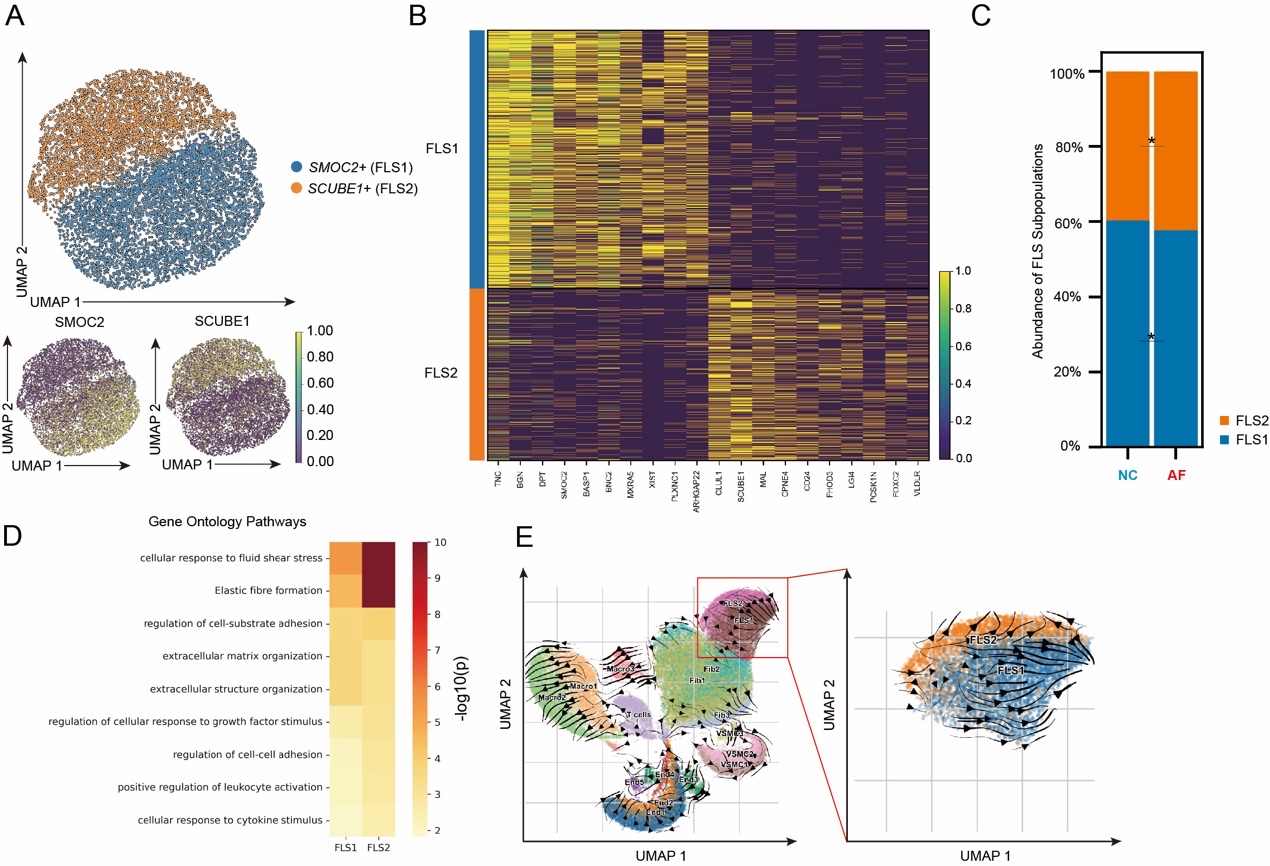


Figure supplement 3. Characteristics of FLS identified in synovial tissues. (A) UMAP of subpopulations of FLS. (B)Differential expressed genes of FLS shown on a heatmap. (C) Abundance of FLS subpopulations compared between the AF group and the NC group. (D) Heatmap of GO analysis for each subpopulation. (E) RNA velocity analysis of FLS.

UMAP, uniform manifold approximation and projection; **P* < 0.05.


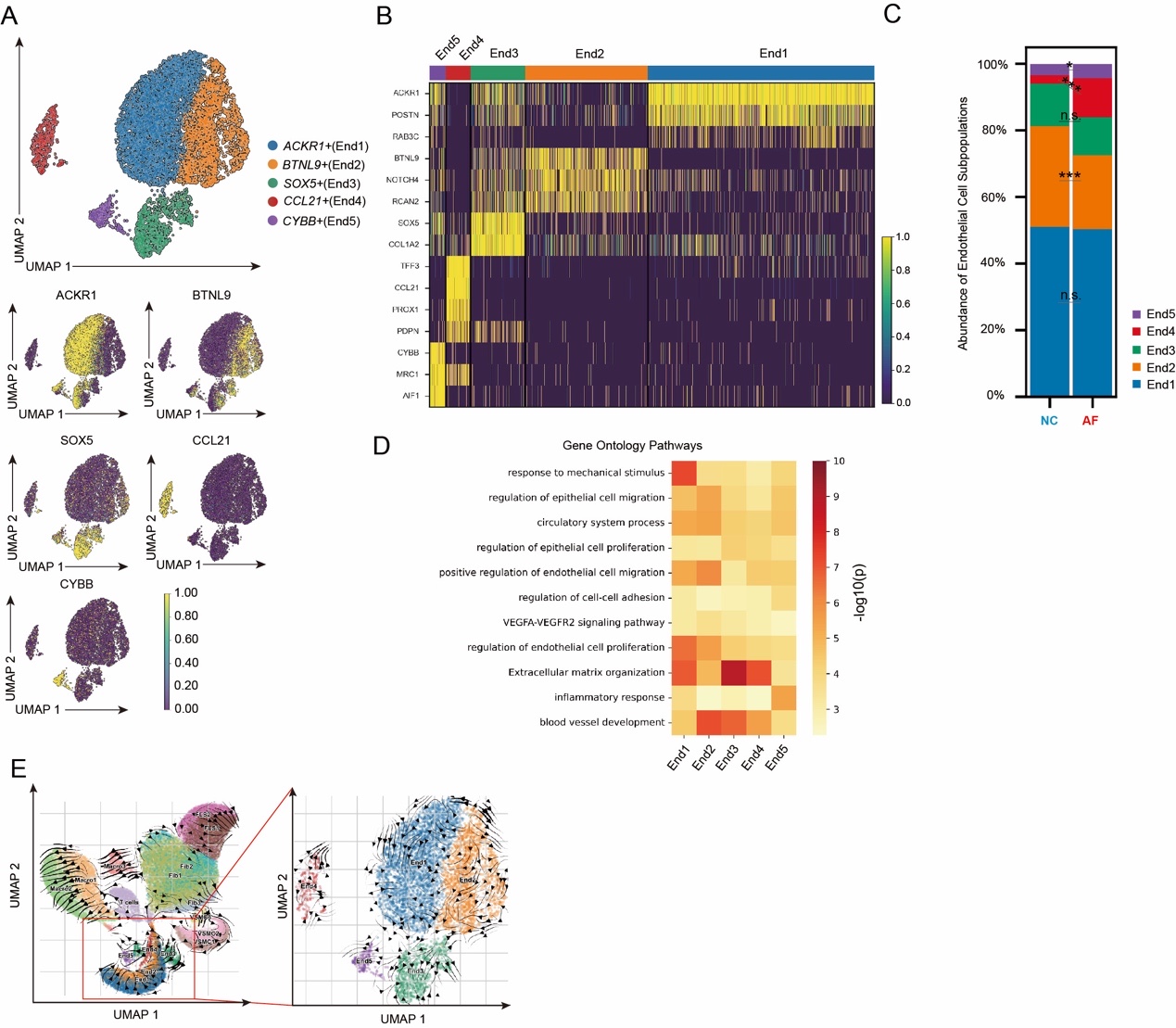


Figure supplement 4. Characteristics of endothelial cells identified in synovial tissues. (A) UMAP of subpopulations of Endothelial cells. (B)Differential expressed genes of endothelial cells shown on a heatmap. (C) Abundance of endothelial cells subpopulations compared between the AF group and the NC group. (D) Heatmap of GO analysis for each subpopulation. (E) RNA velocity analysis of endothelial cells.

UMAP, uniform manifold approximation and projection; **P* < 0.05; *** *P* < 0.001; n.s., no significance.


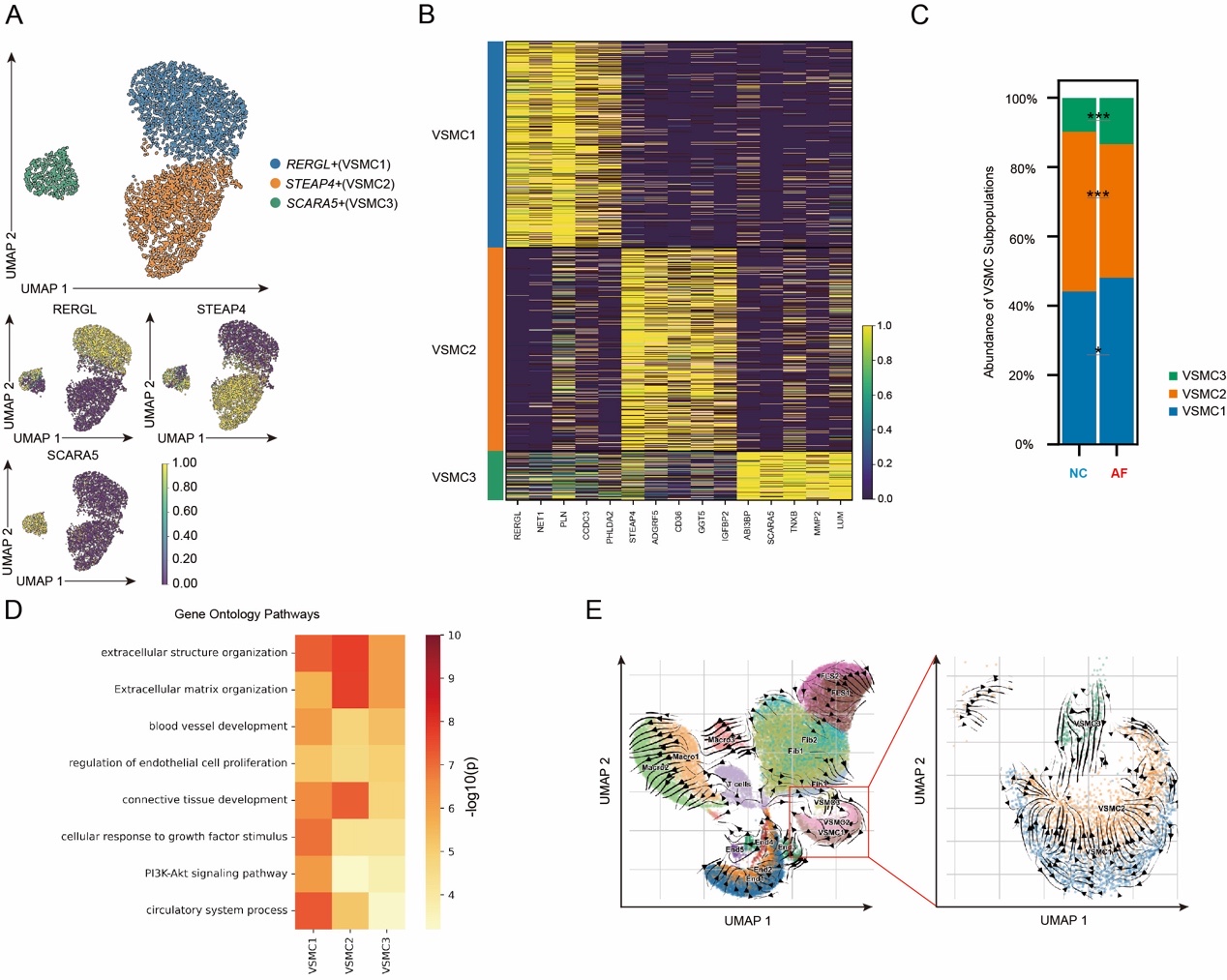


Figure supplement 5. Characteristics of VSMCs identified in synovial tissues. (A) UMAP of subpopulations of VSMCs. (B)Differential expressed genes of VSMCs shown on a heatmap. (C) Abundance of VSMC subpopulations compared between the AF group and the NC group. (D) Heatmap of GO analysis for each subpopulation. (E) RNA velocity analysis of VSMCs.

UMAP, uniform manifold approximation and projection; **P* < 0.05; *** *P* < 0.001.


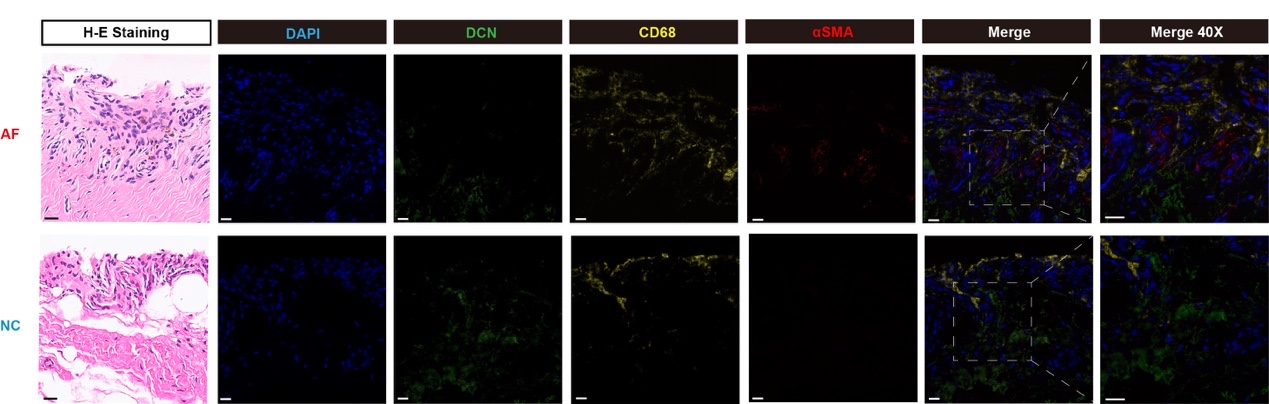


Figure supplement 6. Representative H-E staining and IF staining of synovial tissue in both AF and NC group. DAPI (blue), DCN (green), CD68 (yellow), αSAM (red) in individual and merged channels are shown. Bar, 20 μ m. The images are captured at a magnification of 20x, with the final column showcasing a higher magnification of 40x to highlight finer details.
